# Assessment of physical, microstructural, thermal, techno-functional and rheological characteristics of apple (*Malus domestica*) seeds of Northern Himalayas

**DOI:** 10.1038/s41598-021-02143-z

**Published:** 2021-11-23

**Authors:** Mehnaza Manzoor, Jagmohan Singh, Adil Gani

**Affiliations:** 1 Division of Food Science and Technology, Sher-e-Kashmir University of Agriculture Science and Technology, Jammu, 180009 India; 2 Department of Food Science and Technology, University of Kashmir, Srinagar, 190006 India

**Keywords:** Plant sciences, Materials science

## Abstract

In this research, two common apple seed cultivars Viz: ‘Golden Delicious’ (GD) and ‘Red Delicious’ (RD) of Northern Himalayan region were characterized for physical, techno-functional, microstructure, thermal, and rheological properties. Seeds showed a significant difference in width, arithmetic, and geometric mean diameters, volume, and surface area. Proximate analysis results revealed that seed flours have high oil content (> 20%) and are potentially rich sources of protein (> 40%). Color analysis of flours indicated their satisfactory whiter color with higher brightness values (L* ˃ 75), resulting from the reduced particle size which allows greater light penetration and relatively lower a* (< 1.5) and b* (< 11) values. Techno-functional attributes including water/oil absorption capacity, emulsifying capacity, and emulsion stability were significantly higher in RD than GD flour. There was also a significant difference in the average particle size of seed flours. Flour micrographs indicated the presence of oval/spherical-shaped starch granules embedded in dense protein matrix while, Differential Scanning calorimeter (DSC) revealed exothermic transition enthalpies for seed flours. Additionally, seed flours depicted high elastic modulus (G′), suggesting their suitability for modifying food texture. It was concluded that apple seeds exhibit significant potential for use in formulating protein-enriched foods while contributing to reducing industrial wastage.

## Introduction

Apple (*Malus domestica*) is one of the most important fruit crops cultivated throughout the temperate regions of the world. The fruit is consumed fresh or processed into juice, concentrate, jam, cider, canned products. Processing, however, generates a huge quantum of pomace which accounts for up to 20–35% share of fresh fruit weight and consists of a mixture of skin/ pulp tissue (94.5%), with a significant amount of seeds (4.1%) and stems (1.1%)^1,2^. Apple seeds are reported to be an excellent source of proteins (38.85–49.55%) and lipids (20.69–24.32%) with linoleic and oleic acids as predominant amino acids^3^. They also contain a considerably high amount of polyphenolics mainly dihydrochalcones (phloridzin), hydroxycinnamic acid, flavan-3-ol, and flavonols which are effective in many aspects of human health, including reducing the risk of obesity and diabetes mellitus^4–6^, help prevent bone loss, inhibit cancer cell growth, enhance memory and life span^5^. Manzano and Williamson^7^ reported that phloridzin, a chalcone derivative, has an antidiabetic effect by inhibiting sodium-linked glucose transporters. Additionally, apple seeds have good commercial potential as promising antioxidants and can inhibit lipid peroxidation and protects the food from unstable reactive oxygen species^6^. Currently, apple seeds are gaining more interest in food formulations and as potential delivery vehicles for the administration of bioactive compounds^5^. The incorporation of defatted apple seed flour in various food systems has been extensively studied. Puric et al.^2^ examined the effect of adding defatted apple seed cake (DASC) in bread making and observed that bread supplemented with 20% DASC exhibited better nutritive value primarily due to increased protein (15.99%) and insoluble dietary fiber (7.21%) content. Also, a relatively higher total phenolic content, best antioxidant activity, and lowest change in freshness were found in bread supplemented with 20% DASC. In another study, Sudha et al.^8^ reported cake making from apple pomace and wheat flour blend at 0–30% to enrich it with total dietary fiber and polyphenols which have antioxidant activity.

**Figure 1:**
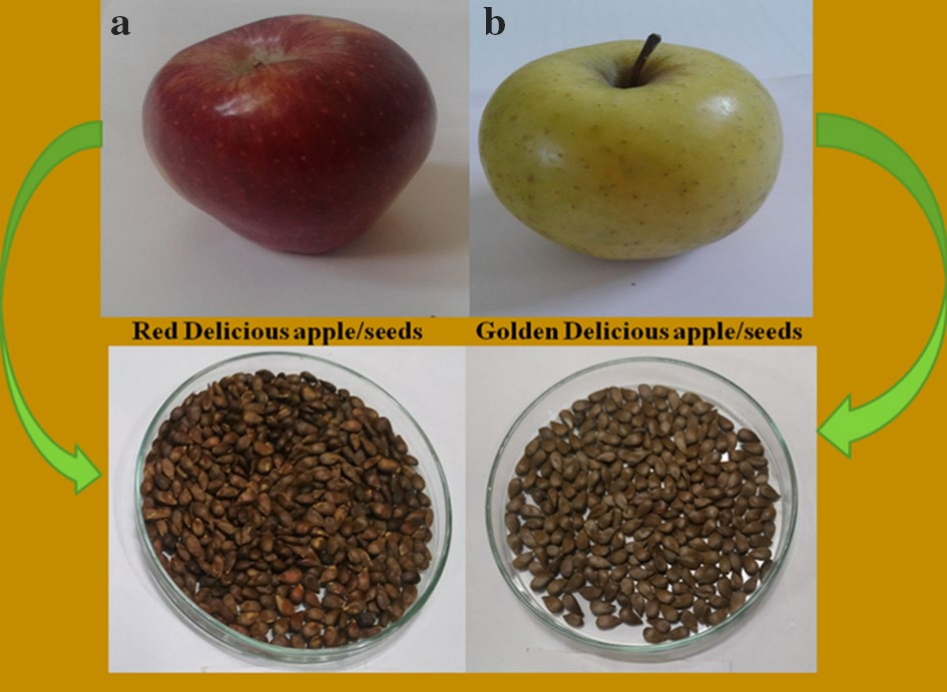
Representative picture of (**a**) Red Delicious apple and seeds, (**b**) Golden Delicious apple and seeds.

Moreover, depending on the variety, apple seeds vary in physical, nutritional, and biological properties. The knowledge regarding basic geometrical properties such as size, shape, volume, density, sphericity, etc., is a useful parameter for designing or modifying machines whose operations are mainly influenced by compressibility and flow behaviour of materials and analyzing the behaviour of product during various processing operations such as harvesting, cleaning, separating, sorting, conveying and griding^9^. Besides physical parameters are the basic quality indicators of seeds that ensure their profitable use in product formation. Variations in morphology including average starch granular size are known to influence the functional properties such as water absorption capacity, swelling power, solubility, thermal and rheological properties of flour obtained from different plant sources. Thus an understanding of the relationship between physiochemical, functional, morphological, thermal, and rheological properties is very important for determining the behaviour of nutrients during processing and their valorization as functional additives in food formulations^10^. Although many studies have determined physical and mechanical characteristics of agricultural materials such as tung (*Aleurites fordii*) seeds^9^, melon (*Cucumis melo* L.) seeds^11^, sunflower (*Helianthus annuus*) seeds^12^, Akee apple (*Bilphia sapida*) seed^13^, oats (*Avena sativa* L.)^10^, finger millet (*Eleusine coracana*)^14^, no such study has been carried out for apple seeds. Besides, there is no study on the thermal, morphological, and rheological properties of apple seed flours. Due to the lack of such studies, apple seeds are rarely used at the commercial level and they generally go to waste. The work was, therefore, focused to determine the basic geometrical, morphological, thermal, techno-functional, and rheological properties of underutilized apple seeds collected from two common varieties ‘Red Delicious’ and ‘Golden Delicious’ (Fig. 1) of the Northern Himalayan origin to feed the growing functional food industry.

## Results and discussion

### Physical characterization of apple seeds

Physical dimensions measured in terms of length, width, thickness, arithmetic, and geometric mean diameter, and shape provide valuable information for differentiating between varieties and/or other grains and in designing material handling and processing machinery. Results of the physical characterization of flour samples are presented in Table 1. The length, width, and thickness of apple seeds varied significantly, with length ranging from 7.69 mm (GD) to 9.43 mm (RD), seed width in a range of 4.02 mm (GD) to 4.87 mm (RD), and thickness in a range of 2.01 mm (RD) to 2.03 mm (GD), respectively. The arithmetic mean diameter and geometric mean diameter of seeds measured were also found to vary significantly and were 4.58 mm, 5.44 mm, and 3.97 mm, 4.51 mm respectively, for GD and RD cultivars. Similar results were obtained on Sabzar, SKO20, and SKO90 oats as reported by Shah et al.^10^ with length ranging from 8.2 to 9.03, width 1.50 to 1.73, and thickness 2.25 to 2.66, and geometric and arithmetic diameters varying from 3.12 to 3.40 and 4.07 to 4.33 mm respectively. Seed surface area, which determines the shape of seed is considered important in heat and mass transfer operations^12^ and its value ranged from 49.57 mm^2^ to 64.19 mm^2^. It was found that the lowest volume was occupied by GD seeds (20.22 mm^3^) compared to RD seeds (29.08 mm^3^). Similarly, sphericity ranged from 48.18% (RD) to 51.72% (GD) indicating a higher resemblance of GD seeds towards a sphere. This can be attributed to the higher moisture content of GD seeds compared to RD seeds. The increase in sphericity with increased moisture content was reported by Malik & Saini^12^ in sunflower seeds. Besides, sphericity and length are inversely related, with round grains resembling more to a sphere than a cylinder. Similar relations were depicted in our study with more spherical grains having the lowest length. However, both varieties depicted non-significant variation in aspect ratio, which indicates the tendency of seeds to roll on a flat surface. Also, the thousand-grain weight, which usually indicates the endosperm content in the seeds, was found to be 44.83 g (GD) and 45.32 g (RD) with no significant difference noticed between cultivars. All such variations could be attributed to the genetic makeup of the seed cultivars and will play an important role in the machine and other post-harvest equipment designing for efficient handling, processing, and storage of materials.

**Table 1:** Geometrical properties of apple seeds (n = 3).

Parameter	RD	GD
Length (mm)	9.43 ± 1.07^a^	7.69 ± 0.43^a^
Width (mm)	4.87 ± 0.15^a^	4.02 ± 0.01^b^
Thickness (mm)	2.01 ± 0.14^a^	2.03 ± 0.13^a^
Geometric mean diameter (mm)	4.51 ± 0.20^a^	3.97 ± 0.11^b^
Arithmetic mean diameter (mm)	5.44 ± 0.39^a^	4.58 ± 0.15^b^
Seed volume (mm^3^)	29.08 ± 3.63^a^	20.22 ± 1.65^b^
Sphericity (%)	48.18 ± 4.06^a^	51.72 ± 2.31^a^
Surface area (mm^2^)	64.19 ± 5.95^a^	49.57 ± 2.96^b^
Aspect ratio (%)	52.05 ± 4.73^a^	52.38 ± 2.91^a^
Thousand-grain weight (wt.g)	45.32 ± 0.47^a^	44.83 ± 0.22^a^

Different superscript on mean values with standard deviation ( ±) in same row indicates statistical difference (p ≤ 0.05).

RD and GD represent Red Delicious seeds and Golden Delicious seeds.

### Chemical composition of seed flour

Table 2 presents the proximate analysis such as moisture, crude fat, protein, and total ash content of ‘Red Delicious’ and ‘Golden Delicious’ seed flour. Moisture content is an important parameter that can help suggest the storage stability of dried products. Higher moisture content tends to shorten the shelf life and result in loss of physical, chemical, biochemical properties, and as well as results in rapid microbial growth^14^. The results for moisture content were found to be 8.17% for RD seed flour and 8.57% for GD seed flour with no significant variation observed among cultivars. These results were agreeable to that reported for Akee apple seed flour (8.05%)^13^. It was also found that RD flour contained significantly high (p ≤ 0.05) crude protein and fat content. The crude protein content was found to be 41.31% (GD) and 47.98% (RD) and was higher compared to other fruit seeds such as kiwi fruit flour (4.75–8.74%)^15^, quinoa flour (13.46%)^16^, watermelon, guava, orange, apricot, paprika and prickly-pear^17^ and also protein-rich flours, such as chickpea (20–24.3%)^18^, cowpea (24.1%) and horse gram flour (22.5%)^19^. This high protein content might be substantial enough to have a decisive effect for supplementing composite flours for the development of nutrient-enriched food products. Regarding protein quality, apple seeds are reported to be rich in sulfur-containing amino acids and appear to be reasonably balanced, with most of the essential amino acids above the FAO/WHO pattern (FAO/WHO, 1993). Concerning lipid content, a significantly highest percentage was found in RD seed flour (25.31%) than GD seed flour (21.47%). These results are comparable to that reported for *L. acidissima* (Wood apple) seed flour (24.9%)^20^ but superior to that reported for quinoa seed flour which has only 5.47% oil^16^. However, total ash content, which is related to minerals, was not found to vary significantly and ranged from 4.35% (RD) to 4.67% (GD). These results were found to be higher than in *C.lanatus* (2.75%) and *L. acidissima* (2.84%) seed flour^20^. Overall, both seed varieties presented a good nutritional value that can contribute to the formulation of protein-enriched functional foods to combat malnutrition.

**Table 2:** Physiochemical properties of flour samples.

Cultivar	Moisture content (%)	Protein (%)	Fat (%)	Ash (%)
RDF	8.17 ± 0.17^a^	47.98 ± 2.04^a^	25.31 ± 0.99^a^	4.35 ± 0.73^a^
GDF	8.57 ± 0.25^a^	41.31 ± 2.52^b^	21.47 ± 1.12^b^	4.67 ± 0.60^a^

Different superscript on mean values with standard deviation ( ±) in same column indicates statistical difference (p ≤ 0.05).

RDF and GDF represent Red Delicious seed flour and Golden Delicious seed flour.

### Flour flowability properties

#### Bulk density, True density, Carr’s index, and Hausner ratio

The bulk density, which indicates the load flour samples can withstand if allowed to rest directly on one another^14^ was recorded to be 0.38 g/mL and 0.60 g/mL for RD and GD cultivars (Table 3). This variation in bulk density can be associated with particle size, with finer particles resulting in higher bulk density since particles orient in a manner to decrease inter-practical space to occupy less space compared to coarse particles. These results can also be validated from particle size distribution and SEM results which depict a larger size of RD cultivar resulting in reduced mass per unit volume occupied by flour sample compared to GD seed flour. The higher bulk density value of GD seed flour can also be attributed to its high moisture content which increases total mass per unit volume occupied by flour sample. The true density was found higher than bulk density with mean measured values of 0.58 g/mL for RD seed flour and 0.69 g/mL for GD seed flour. For good flowability of powdered samples, bulk and true density values should be close enough to have a lower Carr index. The Carr compressibility index of RD and GD seed flour was found to be 34.51% and 37.70% while the Hausner ratio was found to be 1.52% and 1.6% respectively (Table 3). Carr index ˃ 25% is considered to be a poor flowability indicator, and ˂ 15% a good flowability indicator^20^. These results are in line with the findings of Sonawane et al.^20^ where bulk and true densities measured were 0.26, 0.28 g/mL and 0.39, 0.47 g/mL, which resulted in compressibility index of 34.17% and 41.27% for *C. lanatus* and *L. acidissima* flour respectively.

**Table 3:** Particle size distribution and average hydrodynamic particle size and flowability properties of flour samples (n = 3).

Parameter	RDF	GDF
**Particle size distribution (μm)**		
Dv(10)	6.26 ± 2.20^a^	2.60 ± 3.910^b^
Dv(50)	7.58 ± 3.80^a^	4.41 ± 2.90^b^
Dv(90)	9.06 ± 1.10^a^	6.12 ± 2.30^b^
Average hydrodynamic particle size (μm)	8.73 ± 4.10^a^	2.97 ± 2.10^b^
Polydispersity index	0.43 ± 2.10^a^	0.12 ± 1.10^b^
**Flowability properties**		
Bulk density (g/mL)	0.38 ± 0.10^a^	0.604 ± 0.09^b^
Tapped density (g/mL)	0.58 ± 0.17^a^	0.69 ± 0.13^b^
Carr’s index (%)	34.51 ± 2.99^a^	37.70 ± 1.62^a^
Hausner ratio (%)	1.52 ± 0.06^a^	1.6 ± 0.04^a^

Different superscripts on mean values with standard deviation ( ±) in same row indicate statistical difference (p ≤ 0.05).

Dv (10), Dv (50) and Dv (90) symbolize the points in the size distribution upto which 10%, 50% and 90% of total volume of material in sample is contained.

RDF and GDF represent Red Delicious seed flour and Golden Delicious seed flour.

### Particle size distribution and polydispersity index

Particle-size distribution is a quality parameter influencing the processing performance of flour samples. Table 3 shows the average hydrodynamic diameter and particle size distribution of flour samples in aqueous suspension. The particle sizes of GD seed flour at 10% (Dv10), 50% (Dv50), and 90% (Dv 90) volume distribution was found to be 2.603, 4.419, and 6.122 μm respectively, which was lower than corresponding values for RD cultivar (6.269, 7.580 and 9.060 μm). Moreover, the average hydrodynamic diameter of GD and RD flour fractions was found to be 2.97 μm and 8.73 μm respectively which indicates that these macromolecules were finely ground. It has already been stated that the difference in particle size distribution among different flours might depend on endosperm structure (hard or soft), the grade of material, endosperm cells, and grinding equipment used^21^. Besides, the polydispersity index which reflects homogeneity of sample based on size was found to be 0.12 and 0.43 for GD and RD seed flours respectively, and around 0.4 indicates narrow size distribution of sample particles with good stability in solution^22^.

### Color

Color is an important quality attribute that immensely influences the consumers' preference and choice of food. The results of the color characteristics (Table 4) of both flours presented satisfactory whiter color with higher brightness values (L*) ˃ 75. The significantly (p ≤ 0.05) higher L* value of GD seed flour may be attributed to its reduced particle size (as also reported from SEM results) as compared to RD seed flour, increasing its surface area and thereby allowing greater reflection of light. The lower a* values in both flour samples which is an indication of greenness also displayed a significant (p ≤ 0.05) difference among varieties with RD seed flour presenting more greenness than GD seed flour. Similarly, b* values which indicate blueness and yellowness ranged from 9.18 to 10.64 with a lower value of yellowness for RD seed flour which may be due to a reduction in pigments during processing. Lightness and yellowness are important color attributes that influence consumers' preference and acceptability of products^14^. These results are comparable to the findings of Kaur & Singh^18^ who studied the variation in color in different varieties of chickpea flour. Such variations in color characteristics may result from differences in inherent colored pigment among the flour varieties, resulting from genetic variations of the seeds.

**Table 4:** Color, thermal properties and techno-functional properties of flour samples (n = 3).

Parameter	RDF	GDF
**Color**		
L*	76.87 ± 0.52^b^	78.93 ± 1.02^a^
a*	0.97 ± 0.01^b^	1.31 ± 0.12^a^
b*	9.18 ± 0.41^b^	10.64 ± 0.37^a^
**Thermal properties**		
Onset temperature (°C)	89.07 ± 2.94^a^	78.95 ± 1.69^b^
Peak temperature (°C)	102.17 ± 2.15^a^	98.37 ± 1.25^b^
Conclusion temperature (°C)	117.53 ± 1.66^a^	108.71 ± 2.49^b^
Enthalpy of gelatinization (J/g)	18.41 ± 0.63^a^	9.78 ± 1.33^b^
**Technofunctional properties**		
WHC (g/100 g)	327.65 ± 1.43^a^	312.58 ± 1.57^b^
OHC (g/100 g)	92.97 ± 2.04^a^	86.76 ± 1.74^b^
EA (%)	79.61 ± 1.43^a^	72.01 ± 0.62^b^
ES (%)	51.81 ± 1.07^a^	46.56 ± 1.17^b^

Results are mean ± standard deviation.

Mean values with different letters in the same row indicate a statistical difference (p ≤ 0.05).

RDF, GDF, WHC, OHC, EA, and ES represent Red Delicious seed flour, Golden Delicious seed flour, water holding capacity, oil holding capacity, emulsion activity, and emulsion stability, respectively.

### Scanning electron microscopy (SEM)

**Figure 2:**
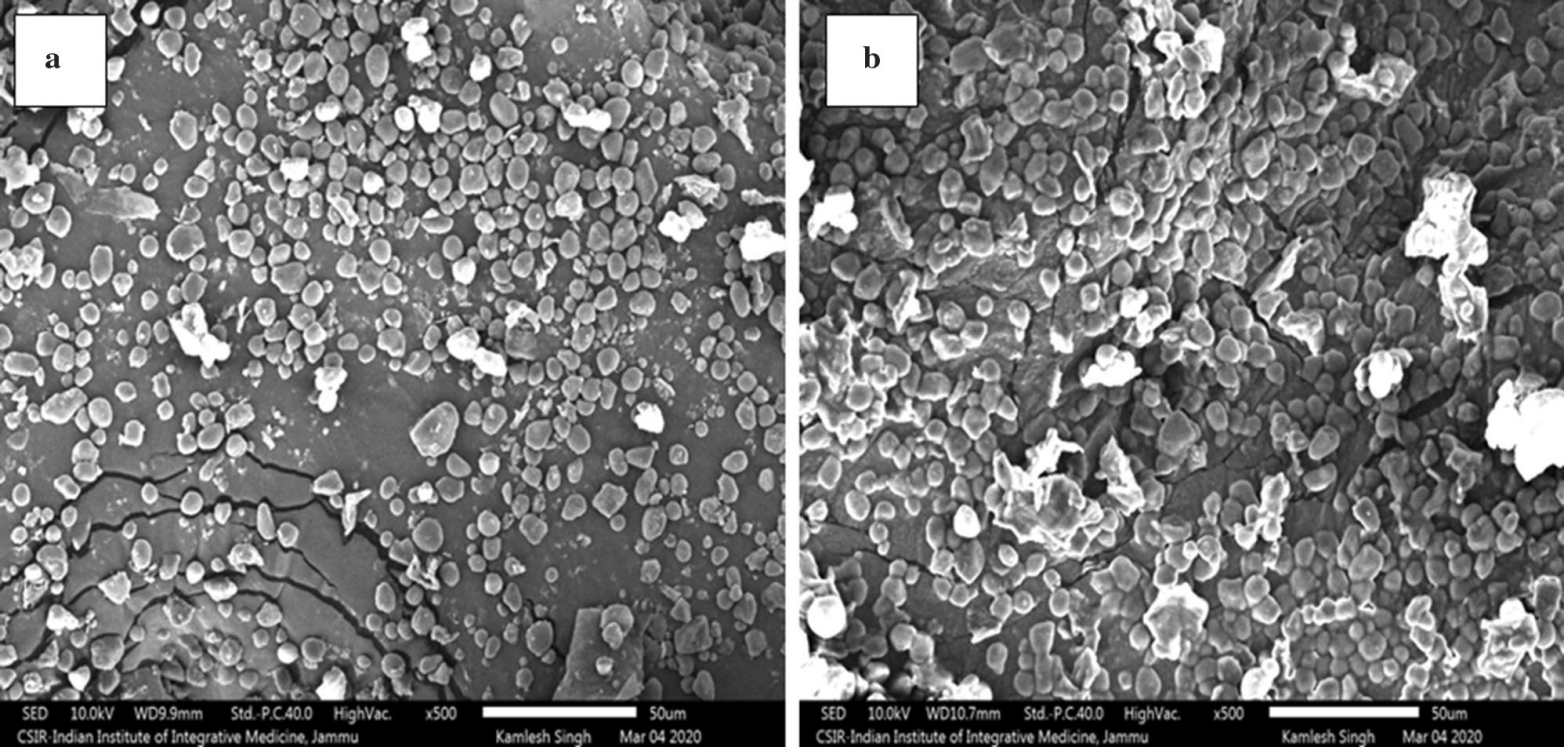
Micrograph of (**a**) Golden Delicious seed flour, (**b**) Red Delicious seed flour at × 500.

The morphological structures of both apple seed flour are depicted in Fig. 2a,b. The SEM morphograph revealed the presence of small and as well as large starch particles of varying shapes and dimensions with some dents/fissures. The starch macromolecules were embedded in a continuous matrix formed by proteins and other non-protein components. The micrograph indicated that starch granules in RD seed flour form clusters and were semi-spherical, oval-shaped, and polygonal with a rough surface and size ranging from 4.56 to 15.27 μm (Fig. 2b). However, GD seed flour (Fig. 2a) depicted spherical and somewhat irregular but smooth-surfaced starch granules with sizes ranging from 2.04 to 14.32 μm very similar to results for oat flour^10^. These changes in conformation as well as the shape and size distribution of starch granules could influence the functionality of seed flours for their further use in functional food development.

### Thermal characterization of apple seed flours

The thermal characteristics of the two seed flours as determined by DSC differed significantly (p ≤ 0.05). The gelatinization transition temperatures (onset temperature, To, endothermic peak temperature, Tp and conclusion temperature, Tc) and enthalpy of gelatinization (∆ H) of two seed flour samples were determined by DSC. The values obtained for ‘Golden Delicious’ and ‘Red Delicious’ seed flours were Tp = 98.37, 102.17 °C, To = 78.95, 89.07 °C, Tc = 108.71, 117.53 °C and ∆H = 18.41, 9.78 J/g respectively (Table 4). Results revealed that gelatinization temperatures of RD seed flour were significantly (p ≤ 0.05) higher than GD variety. As already stated, gelatinization transition temperatures vary with botanical species and usually differ with shape, size, and the internal arrangement of starch fractions within granule^18^. At the same time, the higher endothermic peak temperature of RD seed flour compared to GD seed flour (p ≤ 0.05) might be attributed to its high proteins, lipids, and fiber content that gets degraded at high temperature^23^. Further, a significant (p ≤ 0.05) difference in the gelatinization enthalpy was also recorded among the two cultivars with RD seed flour having a generally lower ΔH value (9.78 ± 1.33 J/g) compared to GD seed flour (18.41 ± 0.63 J/g) which indicates its low energy requirement for crystallite melting. It may be due to a greater number of double-helical regions within GD granules which require more energy for breaking hydrogen bonds between glucan chains for complete starch gelatinization. Ren^24^, reported that particle size also affects ∆H value suggesting that flour with small particles results in complete protein denaturation and starch gelatinization which is consistent with the SEM results of this study.

### Techno-functional properties of flour samples

The techno-functional properties that have been categorized as non-nutritive food characteristics with a critical contribution in improving the existing functionalities and helping in new product development were also investigated with results depicted in Table 4.

#### Water holding capacity

Water holding capacity determines the ability of a product to hold water depending on the presence of polar/ hydrophilic proteins and polysaccharides^18,25^. The results depicted a significantly (p ≤ 0.05) higher water holding capacity (WHC) of RD seed flour (327.65 g/100 g) compared to GD seed flour (312.58 g/100 g). Earlier, El-safy et al.^17^ reported comparable results for the Egyptian variety of apple seed flour (3.58 g/g), whereas lower values were found for watermelon, guava, orange, apricot, paprika, and prickly-pear. The existence of higher protein content in flour samples (Table 1) might contribute to their higher WHC. Since it has been reported that the presence of several hydrophilic proteins especially polar amino acid residues bind subsequently with more water molecules resulting in higher water absorption^19^.

#### Oil holding capacity

Regarding oil absorption capacity (OHC), the values 92.97 g/100 g and 86.76 g/100 g observed in the present work for RD and GD seed flour, respectively are low compared to values given for the Egyptian variety of apple seeds flour^17^. This variation in fat absorption among two cultivars can be attributed to variation in protein concentration and hydrophobicity of proteins which in turn determine its degree of interaction with oil and water^26^.

#### Emulsion activity and stability

Emulsification activity (EA) characterizes the capacity of flour samples to form stable emulsion via the interaction of polar and non-polar proteins with oil droplets at the oil–water interface. Therefore, more is protein content more will be adsorption ability towards oil. The EA was recorded to be 79.61% for RD seed flour and 72.01% for GD seed flour with a significant (p ≤ 0.05) difference observed among cultivars. Also, emulsification stability (ES), which indicates if the globular proteins present in the flour sample can prevent deformation of the emulsion system under shear stress conditions^19^ was found to have a higher value for RD seed flour (51.81%) than GD seed flour 46.56% after 60 min (Table 4). A comparatively lower EA of 47.58% and 40.60% and ES of 45.22% and 11.48% were reported for *C. lanatus* and *L. acidissima* seed flours respectively^20^. Therefore high EA and ES of apple seed flours will render them more suitable for surface adsorption for use in stabilizing colloidal food systems and in processed meat products such as sausages^20^.

### Dynamic rheology

**Figure 3:**
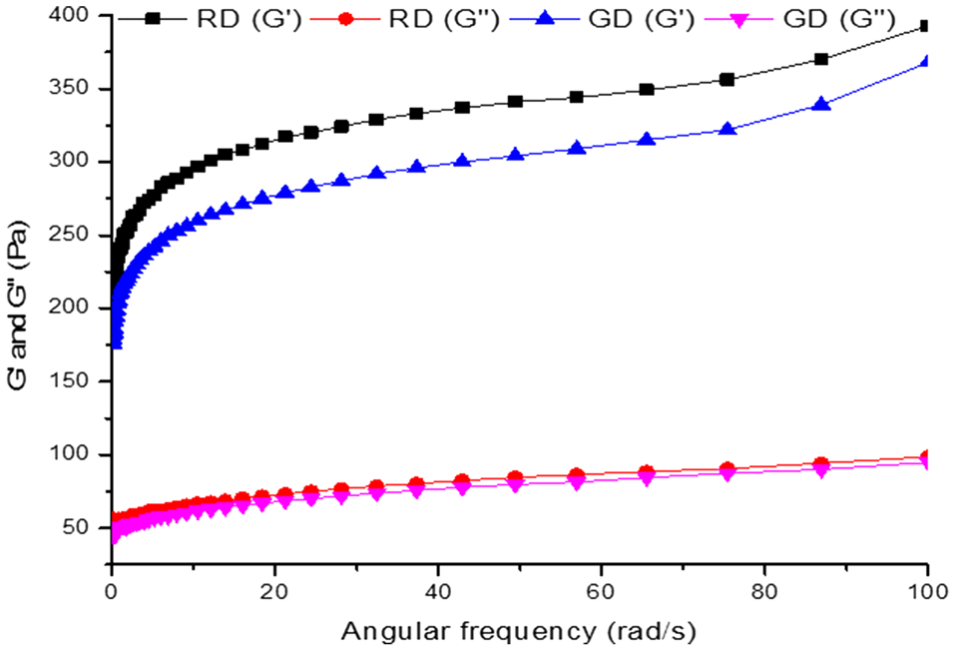
Loss (G″) and storage (G′) of Red (RD) and Golden Delicious (GD) seed flour.

The difference in rheological behaviour (visco-elastic properties) of the two flour samples was expressed in terms of loss moduli G′′ and storage moduli G′. Variation curves of G′′ and G′ as a function of oscillatory frequency are depicted in Fig. 3. The mechanical spectra of both samples obtained in this study displayed consistently higher G′ than G′′ throughout the frequency range studied, which indicated typical viscoelastic behaviour with a predominant solid or elastic (gel-like) character. Generally, higher G′ is an indication of weak gel behaviour. Lower viscous modulus G′′ compared to elastic modulus G′ indicates that flour samples are of good quality which could lead to higher bread loaf volume. Interestingly, Jhan et al.^26^ reported a similar trend of dynamic moduli of pearl millet used in their studies. Moreover, RD seed flour exhibited a pronounced increase in visco-elastic behavior compared to the GD variety. This could be due to the higher protein percentage in RD seed flour which favours protein aggregations resulting from increased disulfide linkage and enhanced interaction of gluten network within the matrix favouring visco-elasticity of the continuous phase. The effect of protein in increasing the G′ and G″ was reported by Marco and Rosell^27^. The results were also consistent with the proximate composition wherein RD and GD seed flours showed higher protein concentrations.

## Conclusion

The study provided a basic understanding of various geometrical, physiochemical, functional, morphological, thermal, and rheological properties of two underutilized apple seed cultivars. The experimental results revealed that apple seeds differ considerably in their dimensional properties which will enable their sorting in different fractions for post-harvest operations. A considerable good amount of protein and lipid content was observed in seed flours which suggest their use in the development of protein-enriched food products with different end uses. Further, flour prepared from RD seeds depicted smaller particle size distribution with high stability to thermal degradation, mostly suitable for various food processing operations. Likewise, a significant difference was observed in techno-functional properties with RD seed flour having significantly higher water/oil holding capacity, emulsification capacity, and emulsification stability than GD seed flour. Moreover, rheological results revealed predominant visco-elastic behaviour of seed flour which could help modify the texture of various foods. Therefore, the exploitation of apple seeds can be a boon for food security while minimizing industrial food wastage and as well contribute to the development of protein-enriched foods to combat malnutrition.

## Materials and methods

### Materials

Apple seeds (‘Golden Delicious’ and ‘Red Delicious’) were procured from the Juice processing industry (FIL Industries Pvt Ltd, Srinagar, J&K, India). The collection of seed samples was in compliance with the institutional guidelines. The samples were cleaned, dried, dehulled, and then stored at 4 °C in airtight containers. All chemicals and reagents used were of analytical grade and purchased from High media Laboratories Pvt Ltd and Sigma Aldrich (USA).

### Sample preparation

The seed flours were prepared following the method described in our previous work^6^.

### Physical characterization of apple seeds

#### Geometric properties

The three principal axial dimensions (mm) including length (L), width (W), and thickness (T) of 100 randomly selected seeds of each variety were measured by digital vernier caliper reading to an accuracy of 0.01 mm.

##### Geometric and arithmetic mean diameter

Geometric mean diameter, Dg (mm), and arithmetic mean diameter, Da (mm) of seeds were calculated using Eqs. (1) and (2) as described by Shah et al.^10^.

1 $${D}_{g}=\sqrt[3]{LWT}$$

2 $${D}_{a}=\frac{LWT}{3}$$

##### Sphericity

The shape of seeds can be determined in terms of sphericity (φ), which is an index of roundness and was calculated by the formula used by Ramashia et al.^14^.

3 $$\Phi =\frac{\sqrt[3]{LWT}}{L}$$

where; L, W, and T are the length, width, and thickness of seeds respectively.

##### Aspect ratio

The aspect ratio (R_a_) was calculated by the equation used by Shah et al.^10^ as:

4 $${R}_{a}=\frac{W}{L}\times 100$$

##### Volume and surface area

Sample volume, V (mm^3^), and surface area S, (mm^2^) was calculated from geometric mean diameter by the following equation:

5 $$ S = \pi \times D_{g}^{2} $$

6 $$ V = \frac{\pi }{6}D_{g}^{3} $$

##### 1000-grain weight

50 seeds were selected randomly and weighed in digital electronic balance (Shimadzu, unic Bloc) of 0.001 g accuracy. The value was then multiplied by 20 to get the weight of 1000 seeds.

### Proximate composition of flour

The moisture (925.10), fat (920.85), ash (923.03), and protein (920.87) contents were determined by the official AOAC method^28^.

### Flour flowability properties

#### Bulk density and tapped density

Bulk density was measured according to the method described by Gani et al.^29^. The tapped density was determined by the same procedure, but the sample in the measuring cylinder was tapped on the bench very carefully until a constant flour volume was observed.

#### Compressibility index and Hausner ratio

The Compressibility index (CI) and Hausner ratio (HR) were calculated as per the formula given by Carr^30^.

7 $$CI=100 \times \left[1\right.-\left.\frac{{\rho }_{b}}{{\rho }_{t}}\right]$$

8 $$HR=\frac{{\rho }_{t}}{{\rho }_{b}}$$

where *ρ*_b_ is flour bulk density and *ρ*_t_ is flour tapered density.

### Particle size distribution and polydispersity index

The particle size distribution of flour samples was measured using a Litesizer 500 laser light scattering instrument (Anton Paar, Australia). 0.01% flour samples were suspended in Milli-Q water (Elix-10, Millipore, Mosheim, France) and sonicated at 40 kHz (15–30 min) for complete dispersion of flour fractions. The measurements were performed at 20 °C at neutral pH.

### Color

The color of the flour samples was determined by Color Flex Spectrocolorimeter (Hunter Lab D-25, Ruston, USA) which was calibrated using a white reference tile. The results were expressed in terms of L* (lightness/brightness), a* (redness/greenness), and b* (yellowness/blueness) values.

### Scanning electron microscopy (SEM)

The microscopic structure of flour samples was analyzed by scanning electron microscope (Zeiss, EVO 50) under a high vacuum. The samples were mounted on a circular aluminium specimen stub using double-sided adhesive carbon tape. After coating vertically with gold–palladium, the samples were photographed at an accelerator potential of 10.00 kV.

### Thermal characterization of apple seed flours

The thermal characteristics of flour samples were studied using Mettler Toledo DSC-1 STAR^e^ System. Samples (3.5 mg) were weighed into aluminum pans and mixed with Milli Q water (8 μL). The pans were sealed hermetically and allowed to equilibrate for 1 h before analysis. The heat rate was 10 °C/min over a temperature range of 20 to 180 °C in a nitrogen atmosphere. An empty platinum pan was used as the reference. From the curve, enthalpy of gelatinization (ΔH), the onset (To), peak (Tp), and end (Tc) temperatures were obtained using the data processing software supplied with the DSC instrument.

### Techno-functional properties

#### Water/oil absorption capacity (WAC/OAC)

The samples (1 g) were poured in a pre-weighed centrifuge tube and mixed with 10 mL of water/oil. The tubes were vortexed for 5 min and then allowed to stand for 20 min at room temperature. Thereafter tubes were centrifuged (5810R, Eppendorf, Germany) at 3000 rpm for 30 min and supernatant (distilled water/oil) decanted and wet residue weighed for determination WAC and OAC.

#### Emulsification properties

Emulsion capacity (EC) and stability (ES) were determined according to the method described by Hussain et al.^25^. Flour samples (0.5 g) were mixed with 5 mL distilled water in centrifuge tubes and vortexed for 30 s. 5 mL oil was added to the suspension, homogenized, and then centrifuged at 1100×*g* for 5 min (Eppendorf, Germany). The emulsification activity expressed in percentage was calculated as:

9 $$\text{Emulsion\, capacity }(\text{\%})= \frac{\text{H}2-\text{H}1}{\text{H}1 }\times 100$$

where H_2_ is the height of the emulsified layer in the tube and H_1_ is the height of total contents before centrifugation.

The emulsification stability was estimated by further heating the emulsion for 30 min at 80 °C and centrifuged at 1100×*g* for 5 min and expressed as:

10 $$\text{Emulsion\,stability }(\text{\%})= \frac{Ht}{H2}\times 100$$

where H_t_ is the height of the emulsified layer after heating and H_2_ is the height of the emulsified layer before heating.

### Dynamic rheology

The rheological analysis was carried out using MCR 102 rheometer (ANTON Par, Austria). The flour suspensions (1% w/v) were prepared with Milli-Q water. The suspensions were homogenized at 1000 × g for 20 min and thereafter equilibrated at room temperature for 5 min. The homogenized samples were subjected to frequency sweep testing from 0.1 to 100 rad/s performed at 25 °C for determining the changes in storage modulus (Gʹ) and loss modulus (Gʹʹ) occurring during the test.

### Statistical analysis

All experiments have been carried out in triplicates and data was expressed as mean ± standard deviation. The results obtained were statistically analyzed by paired student *t-*test. The statistical difference was determined with p ≤ 0.05.
